# Identification of offensive language in Urdu using semantic and embedding models

**DOI:** 10.7717/peerj-cs.1169

**Published:** 2022-12-12

**Authors:** Sajid Hussain, Muhammad Shahid Iqbal Malik, Nayyer Masood

**Affiliations:** Department of Computer Science, Capital university of Science and Technology, Islamabad, Pakistan

**Keywords:** Identification, Offensive langauge, Natural language processing, Urdu, Semantic, Emebedding model, word2vec, TF-IDF

## Abstract

Automatic identification of offensive/abusive language is very necessary to get rid of unwanted behavior. However, it is more challenging to generalize the solution due to the different grammatical structures and vocabulary of each language. Most of the prior work targeted western languages, however, one study targeted a low-resource language (Urdu). The prior study used basic linguistic features and a small dataset. This study designed a new dataset (collected from popular Pakistani Facebook pages) containing 7,500 posts for offensive language detection in Urdu. The proposed methodology used four types of feature engineering models: three are frequency-based and the fourth one is the embedding model. Frequency-based are either determined by the term frequency-inverse document frequency (TF-IDF) or bag-of-words or word n-gram feature vectors. The fourth is generated by the word2vec model, trained on the Urdu embeddings using a corpus of 196,226 Facebook posts. The experiments demonstrate that the stacking-based ensemble model with word2vec shows the best performance as a standalone model by achieving 88.27% accuracy. In addition, the wrapper-based feature selection method further improves performance. The hybrid combination of TF-IDF, bag-of-words, and word2vec feature models achieved 90% accuracy and 97% AUC. In addition, it outperformed the baseline with an improvement of 3.55% in accuracy, 3.68% in the recall, 3.60% in f1-measure, 3.67% in precision, and 2.71% in AUC. The findings of this research provide practical implications for commercial applications and future research.

## Introduction

The advancement in communication technologies has brought geographically scattered people of the world closer to each other, thus forming new virtual societies (Torkey et al., 2021). Popular social websites such as Facebook, Twitter, YouTube, Instagram, *etc*. have provided new forms of social interaction among people. These websites are so popular that the number of active users is expected to reach 3.43 billion by 2023 (Statista, 2021). Among them, Facebook is the first one that surpassed 1 billion active users monthly and ranked first among popular social websites (Statista, 2021). The majority of people are using these platforms to express their feelings and share their thoughts. However, some users exploit the anonymity provided by these platforms by posting offensive posts and comments.

The use of impolite words while using any form of social media is called offensive language (Culpeper, 2011). It has been usually used to insult people regarding their religions, races, ethnicities, disabilities, and gender (Razavi et al., 2010). Offensive language in the form of cyberbullying (Waseem et al., 2017), hate speech (Davidson et al., 2017), and harassment (Yin et al., 2009) has become a serious problem, affecting many internet users. It is evident from the events that occurred in the world that social websites are easy tools for propagating offensive language, that is harmful to our societies. There is a strong connection between offensive language and actual hate crimes against various communities. The ethnically motivated violence against Muslims in Myanmar and the Pittsburgh synagogue shooting, are examples of such incidents. Therefore, it becomes essential to spot it in advance, to be able to take some preventive measures. Furthermore, this advanced spotting of offensive language activity on social media must be automatic. It is not possible to do this critical task manually on such a huge scale. This will ensure the safety of online communities and individuals.

Urdu language usage is rapidly increasing because social media websites are providing localization facilities to their users. The statistics (alphapro.pk) show that there are approximately 35 million active users of Facebook in Pakistan, and this number increases at the rate of 17% annually. The Urdu language draws its vocabulary and grammatical structure from Arabic, Persian, Turkish, and Sanskrit languages. It derives its vocabulary and unicode characters from these languages, thus special care is needed to distinguish these characters (Naseem & Hussain, 2007). In addition, it has a different writing style, from right to left, and has more phonic sounds than all of the above-mentioned languages. Also, there is a lack of standardization of language writing rules. The most common styles in the Urdu language are Nasakh and Nastalique (Sarfraz, Dilawari & Hussain, 2003). Each has its own rule. In Urdu, a character can acquire four different shapes *i.e.*, initial, middle, isolated, and final position in a connected sequence, for example, Urdu letter 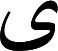 has four shapes ( 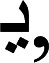 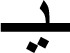, 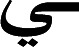, and 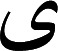). The deficiency of a single standard rule creates a lot of difficulties in text tokenization and language modeling *i.e.*, unigram, bigram, or trigram. In addition, Urdu compound words consist of two or three meaningful words. Literature also reported challenges in stemming, such as stemming the infixes, ambiguous affixes, stemming errors, and stemming the plurals. In addition, Urdu has 40 distinct alphabets. Due to its complex morphological and grammatical structure, few prior studies worked on it and a lack of available datasets are reported.

Most of the prior works on offensive language detection are in the English language but some studies have addressed this issue in other languages, like Danish (Sigurbergsson & Derczynski, 2019), German (Wiegand, Siegel & Ruppenhofer, 2018), and Italian (Bosco et al., 2018). Recently, Akhter et al. (2020) proposed an approach for offensive language detection in Urdu and Roman Urdu using YouTube comments. They have used word n-grams and char n-grams features but ignored more recent and effective feature extraction approaches, like bag-of-words, TF-IDF, and some sort of embedding/contextual dimensions. In addition, their data set size is very small (2,151 instances). Therefore, it is needed to explore new features on a comparatively large dataset so that findings could be generalized.

To overcome these limitations, we have gathered a larger collection of Urdu posts and comments from public Facebook pages. These pages are of different Pakistani media newsgroups like religious groups, political party groups, and popular bloggers, thus covering many categories. Offensive language could be in various forms; therefore, it is necessary to separate offensive posts and comments from others. After filtering, we got them annotated by five experts following a set of guidelines (see Appendix A), containing 7,500 posts in total, 3,750 of them are offensive and 3,750 of them are not. After that, frequency-based and word-embedding features are extracted, followed by building a binary classification model using five popular machine-learning algorithms. Word n-gram, Bag of Words, TF-IDF, and word2vec feature extraction methods are explored.

To develop an effective identification model, we address the following research questions in this study:

RQ1: How to detect offensive Urdu language on Pakistani social media platforms?

RQ2: What are the most contributing features of frequency-based and word embedding types, while using them as standalone as well as hybrid combinations, for offensive language detection?

In summary, the main highlights of the paper are given below:

1. To the best of our knowledge, the first offensive language detection dataset in Urdu, data extracted from popular Pakistani Facebook pages consisting of 7,500 instances and annotated by domain experts following a given set of guidelines.
2. This article presents an ensemble model-based offensive language detection framework for the Urdu language.
3. To the best of our knowledge, the embeddings of word2vec for the Urdu language are designed first time, using a corpus of 196,226 Facebook posts for offensive language detection.
4. The comparison of ML techniques reveals that voting based ensemble model demonstrated the best performance.
5. The proposed model outperformed the baseline with an improvement of 3.55% in accuracy, 3.68% in recall, 3.60% in f1-measure, 3.67% in precision, and 2.71% in AUC.
6. The wrapper feature selection method further improves the performance significantly by achieving a threshold of 90% in accuracy, and 97% in AUC.
7. The comparison between features reveals that word2vec as a standalone model demonstrated the best performance for offensive language detection.
8. The proposed model could be helpful for real-time applications in the Urdu language and its findings could benefit social media users and owners.

The rest of the article is organized as follows: Section 2 describes prior works in offensive language detection and the research gap. Then, Section 3 explains the steps of the proposed pipeline in detail. After that, Section 4 presents various experiments and results. Discussion on results and their implications are discussed in Section 5. Section 6 presents the conclusion and future directions.

## Related Work

Offensive language is the expression of hatred, expressed verbally ranging from simple profanity to much more severe types. The uninhibited behavior in computer-mediated communication is an early concern when the internet started. In 1992, Collins (1992) explored the concept of flaming in computer-mediated communication. In recent years, the computer linguistic community has started to give attention to offensive language detection, in online social media, due to its popularity and large usage. Most of the prior studies used Twitter for corpus creation, while some studies have also used Facebook and YouTube as data sources. Since one of our goals is to create an annotated Urdu language corpus for offensive language, therefore, we have provided a brief overview of studies about corpus collection and annotation with some review about classification methods for identifying offensive language detection.

In 1997, Spertus (1997) used abusive/hostile messages or flame terms for offensive language identification and applied data-driven methods to automatically detect these messages. He combined syntax and semantic features at the sentence level, to create 47-element feature vectors using 720 messages. For classification, he has used a decision-tree generator that correctly categorizes 64% flame messages and 98% non-flame messages. People may personally attack each other using hostile or abusive language when writing emails or in newsgroups. Later, in 2002, Martin (2002) hypothesized that flames are easy to recognize because of their extreme nature and developed an annotated corpus of 1,140 messages, collected from the Usenet newsgroup.

Later, Razavi et al. (2010) used Martin’s dataset and the natural semantic module (NSM) organization log files dataset, to create an automatic flame detection procedure. They extracted features at different levels and used multilevel classification for flame detection using an Insulting and Abusing Language Dictionary. As detecting online harassment is a challenging task, therefore, Yin et al. (2009) developed an abusive language detection model to find online harassment by extracting TF-IDF, n-gram, sentiment, and contextual features. In online communication, verbal abuse is a serious problem, and detecting and removing blacklist words are very important. To address it, Yoon, Park & Cho (2010) proposed a profanity filtering system in the Korean language to filter phoneme-modified profane words using phoneme-based string alignment. They used a lexicon of 9,300 prototype vulgar words for experiments.

On the other end, cyberbullying is the use of technology to bully someone. Reynolds, Kontostathis & Edwards (2011) used a machine-learning approach to detect language patterns used by bullies and their victims and developed rules for automatically detecting cyberbullying. They collected data from the website ‘Formspring.me’, which was labeled by Amazon’s Mechanical Turk and their model achieved 78.5% accuracy. It is a fact that languages on social media are highly unstructured, informal, and misspelled, that’s why offensive language detection models cannot accurately detect offensive language. Chen et al. (2012) used lexical syntactic features to detect offensive language and identified offensive users with enhanced accuracy. They achieved 98.24% precision and 94.34% recall at the sentence level and 77.9% precision and 77.8% recall at the user level.

Until 2013, most researchers used textual features to detect online cyberbullying, ignoring contextual features. Later, Dadvar et al. (2013) were the first to use contextual features (profile information and user characteristics) to improve the performance of cyberbullying detection. Their dataset consisted of 4,226 comments from 3,858 distinct YouTube users and was manually labeled. They hypothesized that the inclusion of user profile information improved the precision and recall to 77% and 55% respectively. The use of curse words in online communication is very common. Using this concept, Wang et al. (2014) studied people’s cursing behavior on Twitter using 51 million tweets from 14 million users. They found that curse words occurred at the rate of 1.15% on Twitter and 7.73% of all the tweets in their dataset consisted of curse words. They concluded that cursing on Twitter is closely related to two negative emotions: sadness and anger.

Hate speech is a special type of offensive language, targeted toward a specific person or group. Gitari et al. (2015) presented a multi-step approach for hate speech classification by creating a lexicon, using hate speech-related semantic and subjectivity features. They concluded that semantic, hate and theme-based features improve both precision and recall. Later in 2016, Silva et al. (2016) conducted the first large-scale study to find hate speech targets on Whisper and Twitter datasets. They used syntactic structures to find hate targets in the posts. Their results showed that on Twitter and Whisper platforms; race, behavior, and physical individuality are the top hate categories. Then Davidson et al. (2017) separated hate speech from instances of offensive language. They used crowdsourcing to label tweets. Their model achieved a precision of 91%, a recall of 90%, and an f1-score of 0.90% using bigram, unigram, trigram with TF-IDF, part-of-speech (POS), and sentiment features.

Later, Waseem et al. (2017) proposed a typology that captures central similarities and differences among hate speech, cyberbullying and online abusive language. They also described annotation guidelines and feature extraction methods. Using this typology, Wiegand, Siegel & Ruppenhofer (2018) divided the offensive language detection problem into subtasks. First, they categorized German tweets as offensive and non-offensive. Second, further annotated offensive tweets as profanity, insult, or abuse. Later Zampieri et al. (2019) enhanced the offensive language detection subtasks problem by proposing a three-level annotation schema, which categorizes offensive language into three categories: (I) offensive language detection (II) categorization of offensive language, and (III) offensive language target identification. Using this schema, they annotated a large English tweets dataset. Until now, most offensive language annotations are available for English and other European languages (Sigurbergsson & Derczynski, 2019; Wiegand, Siegel & Ruppenhofer, 2018; i Orts Ò, 2019; Del Vigna et al., 2017; Pitenis, Zampieri & Ranasinghe, 2020). From the literature, we also found some offensive language detection studies in Arabic (Mubarak et al., 2020), Indonesian  (Ibrohim & Budi, 2018), Hindi (Kumar et al., 2018), Amharic (Yimam, Ayele & Biemann, 2019) Turkish (Çöltekin, 2020), and Roman Urdu (Rizwan, Shakeel & Karim, 2020).

Some studies such as Saha et al. (2021) provided an exhaustive exploration of different transformer models in three low-resource languages (Tamil, Kannada, and Malayalam), and presented a genetic algorithm technique to ensemble different models. Then Husain & Uzuner (2021) investigated the effect of transfer learning across different Arabic datasets and concluded that there is a limited effect of transfer learning on the performance of the classifier, particularly for highly dialectic comments. Similarly, Vargas et al. (2021) provided a new approach for offensive and hate speech detection by incorporating an offensive lexicon, for the Brazilian Portuguese language, validating their approach for both offensive and swearing linguistic expressions.

We summarize the prior literature on offensive language detection in Table 1. By looking at the language column, we can observe that most of the prior works in offensive language detection are in resource-rich languages, *i.e.*, English, European, and a few others like Arabic, Indonesian and Amharic, *etc*. In contrast, Urdu is a resource-poor language and there is only one work presented in the literature on offensive language in Urdu (Akhter et al., 2020). This work used a small dataset that is collected from popular news (ARY Digital) YouTube webpage. The dataset contains 2,151 instances in total. Moreover, the features used to detect offensive language were very basic, *i.e.*, word n-grams, char n-grams, and their combinations. We observe the following gaps in the literature:

**Table 1:** Summary of prior works on offensive language detection in different languages.

**Platform**	**Language**	**Features**	**Classifiers**
750 English messages (Wiegand, Siegel & Ruppenhofer, 2018)	English	Syntax, semantic	Decision-tree
Usenet news, NSM dataset (Statista, 2021)	English	Abusive Language Dictionary (IALD)	Naïve Bayes
Kongregate, Slashdot and Myspace dataset (Waseem et al., 2017)	English	Content, contextual, sentiment, TFIDF	libSVM
YouTube (Sarfraz, Dilawari & Hussain, 2003)	Urdu	Word-n-gram, character n-gram	LogitBoost, Simple Logistic
9,300 Korean messages (Akhter et al., 2020)	Korean	Phoneme based string alignment	R*-tree based searching algorithm
Formspring. Me (Collins, 1992)	English	Lexicon based	C4.5 decision tree
YouTube (Spertus, 1997)	English	Lexical Syntactic Feature (LSF)	Naive Bayes, SVM
YouTube (Martin, 2002)	English	Content-based features, User-based features	SVM
Twitter (Yoon, Park & Cho, 2010)	English	Unigram, Bigram, LIWC dictionary	Binary classifiers
100 blog postings from 10 different websites (Reynolds, Kontostathis & Edwards, 2011)	English	Subjectivity and semantic features	SVM
Twitter, Whisper (Chen et al., 2012)	English	Semantic, lexicon based	SVM
Reddit, Facebook (Davidson et al., 2017)	Danish, English	Bag-of-words, unigrams, word2vec	Logistic regression, Learned-BiLSTM, Fast-BiLSTM, AUX-Fast-BiLSTM
Twitter (Yin et al., 2009)	German	word2vec, fast text	CNN, LSTM
Twitter (Wang et al., 2014)	Spanish, English	Term Frequency (TF),	SVM
Facebook (Gitari et al., 2015)	Italian	Morpho-syntactical features, sentiment polarity, word embedding lexicons	SVM, LSTM
Twitter (Silva et al., 2016)	Greek	TF/IDF, unigrams, bigrams, POS and dependency relation tags	GRU, LSTM
Twitter (i Orts Ò, 2019)	Indonesian	Word n-gram, char n-gram	Naive Bayes, SVM, Random Forest
Twitter (Pitenis, Zampieri & Ranasinghe, 2020)	Amharic	Unigram, Bigram, Trigram	SVM, LSTM
Twitter (Mubarak et al., 2020)	Turkish	Character and word n-grams	SVM
Twitter (Ibrohim & Budi, 2018)	Roman Urdu	BERT, Fast Text	LSTM, CNN

• Most of the datasets used by prior studies are not publicly available.
• There is only one study on offensive language detection in Urdu  (Akhter et al., 2020).
• The Urdu dataset used by Akhter et al. (2020) is very small consisting of only 2,151 instances.
• Lack of appropriate feature engineering: Most of the studies used character n-grams and word n-grams lexical features, Akhter et al. (2020) also used these two.
• Lack of comparison between ML models: Most of the studies used one or two basic machine learning techniques, and have no comparison of simple and ensemble ML models to select the best model for this task.

Therefore, our research contributes in these directions by developing a comparatively large dataset in Urdu, comparing the performance of lexical and embedding features, and comparing basic machine learning and ensemble models to assess their performance

### Framework methodology

**Figure 1:**
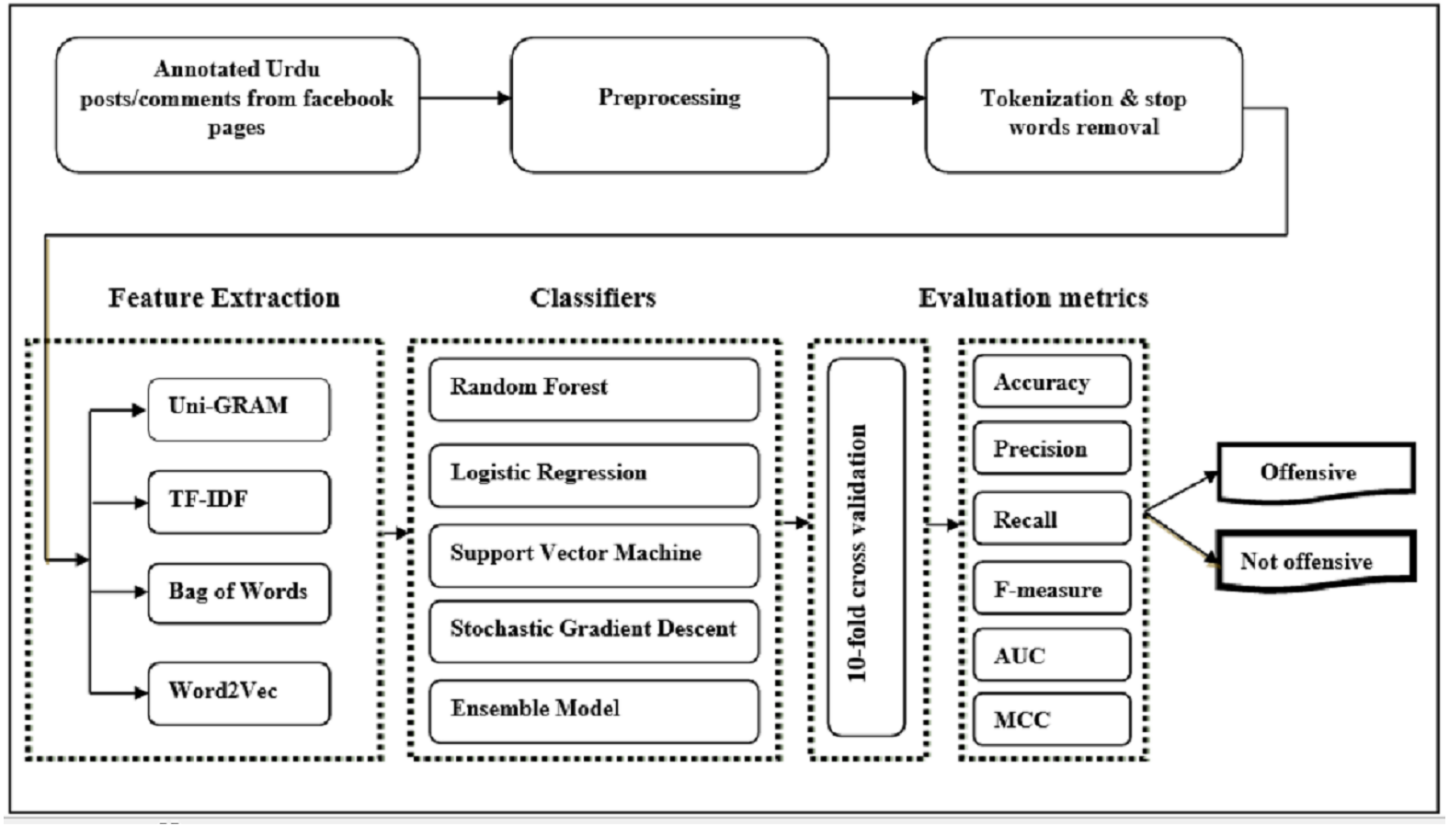
Pipeline of the offensive language detection model.

Social media text is usually in unstructured form and has a wide variety of visualization formats depending upon the specific platform. It is very hard to observe the offensive language in Urdu using only char and word n-gram feature models. Therefore, we use TF-IDF, bag of words, and word2vec feature extraction models in comparison with word n-gram and char n-gram feature models. The pipeline of the proposed offensive language detection model in the Urdu language is presented in Fig. 1. It takes annotated dataset (posts/comments from Pakistani public pages of Facebook) as input, and preprocesses it by removing punctuation marks, white spaces, accents, and inconsistencies from Urdu text. It then tokenizes Urdu text to further prepare it for feature extraction. After tokenization and stop words removal, features are extracted using word unigram, bag of words, TF-IDF, and word2vec extraction methods. Then state-of-the-art classifiers, evaluation metrics, and a 10-fold cross-validation method are used for experimentation. The outcome of the framework is the binary label (offensive or not offensive) of the post.

### Problem formulation

Offensive language detection is the binary classification problem, formally described as follows.

Suppose we have a collection of Facebook posts (P_1_, P_2_, …P_n_) and their corresponding vectors $ \left( {\mathrm{X}}_{1},{\mathrm{Z}}_{1} \right) , \left( {\mathrm{X}}_{2},{\mathrm{Z}}_{2} \right) ,\ldots \left( {\mathrm{X}}_{\mathrm{n}},{\mathrm{Z}}_{\mathrm{n}} \right) $ The variable n represents the total number of posts; *X*_*i*_ is the feature vector related to the post P_i_(X_i_ ∈ R^T^, R^T^refers to the total number of features) and Z_i_ ∈ offensive, not offensive. To classify whether a Facebook post *P*_*i*_ is offensive or not, a predictive function is defined as follows.

(1) \begin{eqnarray*}{\mathrm{Z}}_{\mathrm{i}}={\mathrm{F}}_{\mathrm{OL}}( \frac{{\mathrm{P}}_{\mathrm{i}}}{{\mathrm{X}}_{\mathrm{i}}} ).\end{eqnarray*}

Where

(2) \begin{eqnarray*} {\mathrm{F}}_{\mathrm{OL}} \left( \frac{{\mathrm{P}}_{\mathrm{i}}}{{\mathrm{X}}_{\mathrm{i}}} \right) = \left[ \begin{array}{@{}c@{}} \displaystyle \gt \mathrm{0},~\mathrm{if} {\mathrm{Z}}_{\mathrm{i}}=\mathrm{1}, \text{Offensive}\\ \displaystyle ~=\mathrm{0}, \mathrm{if} {\mathrm{Z}}_{\mathrm{i}}=\mathrm{0}, \mathrm{Not}-\text{Offensive} \end{array} \right] .\end{eqnarray*}

**Objective function:** Our goal is to learn a predictive function that helps to predict whether a post is offensive or not so that future instances can be classified correctly.

### Dataset preparation

Here, we present the process of data collection and annotation to create the offensive language dataset in Urdu, and describe the statistics of the resulting dataset.

#### Domain selection & data collection

We use Facebook’s graph application programming interface (API) to collect posts/comments from Facebook pages. To build a dictionary of seed words, initially, a manual list of offensive words in Urdu is designed. This list is then used to search for other words and keywords used in Facebook posts as offensive. After searching for posts containing these words, the Facebook posts were manually inspected, and more phrases and words are identified. This ended up with enough keywords/words being used as offensive. Some keywords contain more than one word, and some contain only one word. It is necessary to disclose that the selection of these words does not relate to their frequency of occurrence in a post. If a word appears once in a Facebook post to offend someone, it is included in the dictionary such as:

• (Rhinoceros mouth) 
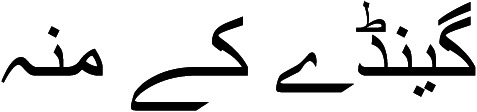

• (Shameless) 
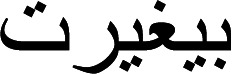

• (Rubbish) 
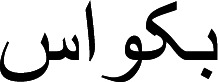

These are examples of offensive keywords. Popular and diverse Facebook pages from different Pakistani newsgroups (religious groups, political parties groups, and popular bloggers) are selected to build the data corpus. We targeted the most popular Facebook pages because these pages disseminate public opinions rapidly. Likewise, the diversity of source pages makes our dataset a good representative of different categories, *e.g.*, religion, politics, *etc*. The sampling criteria and metrics used to select a public page from chosen categories are given below:

1. The number of followers and likes should be greater than 30,000, allowing more active public pages to be selected from categories.
2. Only those pages are selected that employ the Urdu language most frequently for posts and comments.

**Figure 2:**
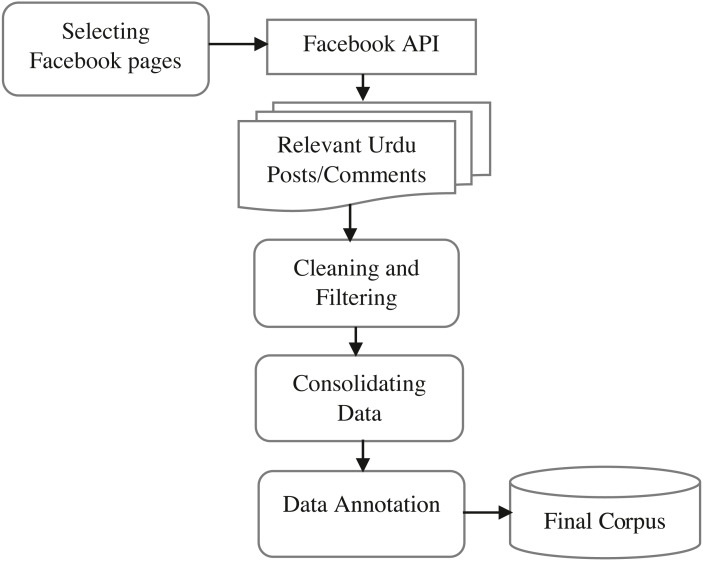
The pipeline of the dataset preparation.

By employing the above criteria, we selected 19 Facebook public pages as described in Table 2. Using the seed word dictionary, we collected Facebook posts/comments containing any of these keywords for 36 months ranging from June 01, 2017, to May 30, 2020. The reason why we choose this period was the general elections held in 2018 in Pakistan and other religious activities. The process of preparation of the dataset with annotation is presented in Fig. 2. Initially, the collection of Facebook posts led to 32,480 instances containing at least one dictionary word. After that, the data was considered for cleaning using the following four steps: (1) since our focus is on the Urdu language, therefore we removed all the posts and comments which are in English, and Roman Urdu, (2) after that, we removed all the punctuation marks, null values, URLs, Emojis, special symbols and numbers from Urdu post/comments because they have no contribution in offensive language detection, (3) we performed normalization on Urdu text to convert homophone variations of Urdu writings to a common symbol *e.g.*, characters ‘ 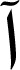
’ and ‘ 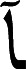
’ are to be replaced by ‘ 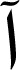
’ and also removes spaces and duplication in the text, and (4) using the Urdu stop word list, we removed stop words from our corpus. After the cleaning steps, the dataset finally led to 12,416 posts. This dataset is further considered for annotation.

**Table 2:** Selected Facebook public pages.

S #	Facebook pages	Before preprocessing		After preprocessing	
		# Of Posts	# Of Comments	# Of Posts	# Of Comments
1	Dunya News	1,000	150,000	460	12,000
2	Samma TV	300	30,000	167	6,600
3	Express News	1,200	100,000	780	22,000
4	Bol News	450	20,000	120	2,500
5	ARY News	800	60,000	333	12,000
6	GeoUrduNews	8,000	105,000	3,000	28,000
7	Urdu Point	2,300	40,000	550	7,500
8	Daily Pakistan	2,800	80,000	880	18,000
9	bbcurdu	5,000	100,000	900	19,000
10	ZemTv	300	18,000	80	1,200
11	PTIOfficial	7,000	110,000	4,400	35,000
12	PPPOfficial	800	45,000	130	8,000
13	PML N Official	1,600	70,000	400	24,500
14	Pervaiz Hood	50	550	10	68
15	JIPOfficial	80	1,000	23	133
16	JUIPK	300	30,000	78	2,500
17	Chingari	400	8,000	80	550
18	marvisirmed	20	1,000	5	125
19	Liberal2016	80	3,500	20	250
	Total	32,480	858,550	12,416	196,226

#### Data annotation

A set of annotation guidelines are designed by considering religious, political, vulgarity, sectarian, and regional contexts to rationalize why a post may be considered offensive or not offensive. We took guidance from prior work (Kumar et al., 2018) to make a set of annotation guidelines (Appendix A).

For annotation of offensive language dataset, we have two available options, (1) crowdsourcing and (2) manual labeling by human experts. We employed the second option. The 12,416 posts dataset and guidelines are then shared with five Urdu experts; among them, two Ph.D. students, one is a master’s student, and the other two are MS-qualified professionals. All annotators are experts in the Urdu language. The majority voting criteria are adopted to decide the final label. As the initial dataset is imbalanced (contains 3,750 offensive posts, and the remaining are not offensive), therefore, for experiments, we draw a sample of 7,500 posts randomly; 3,750 are offensive and 3,750 are not offensive. Regarding data statistics, we observed 57.2%, 31.9%, and 21% annotation agreements among five, four, and three annotators, respectively.

#### Data preprocessing

Pre-processing is an important task to prepare the input Urdu text for classification using several steps such as normalization of text, segmentation of Urdu words, spell correction, tokenization, and stop word removal from the text. We performed normalization to convert homophone variations of Urdu writings to a common symbol *e.g.*, characters ‘ 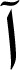
’ and ‘ 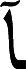
’ are to be replaced by ‘ 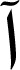
’ removal of spaces and duplication in the text. Then segmentation is used to find the boundaries among the Urdu words. Due to the morphological structure of the Urdu language, we cannot use space to specify the boundary between two words. The space-omission and space insertion are two main challenges related to Urdu word segmentation. After that, tokenization is performed and there are two methods, one is based on punctuation marks and the other is based on white spaces. Stop words are those words that have no impact on text classification therefore stop words are removed using the Urdu stop word list to prepare the text for classification.

### Features extraction

Feature extraction is the most important step in any natural language processing task (NLP). It has been observed that whenever irrelevant features are used, then it may lead to misclassification. Therefore, to design an effective offensive language detection model for the Urdu language, we consider the following state-of-the-art features:

#### Word N-grams

N-grams have been extensively employed in NLP and various text-mining tasks (Langkilde & Knight, 1998). They are also called lexical bundles or multi-word expressions (Csomay, 2013) or a set of co-occurring words within a given window. N-gram is usually a sequence of N words in each sample of text. The sequence may be phonemes, syllables, letters, words, or base words. They always predict the occurrence of a word based on the occurrence of N-1 prior words. As our task is related to NLP, therefore we have used the word n-gram model for Urdu offensive language detection and have extracted unigrams, bi-grams, and trigrams from our text.

#### Bag Of words

In information retrieval and NLP tasks, the bag-of-words method is commonly used as a vector representation for document classification. It transforms text into fixed-length vectors by counting how many times each word appears in the text, also called vectorization (Zhang, Jin & Zhou, 2010). In this method, instead of using predefined words, a domain of corpus is created from the training data to capture opinion words. After designing the corpus, the frequency of each word in the sentence is calculated and this frequency is used as a feature for training a classifier.

#### TF-IDF

TF-IDF is often used as a weighting factor (https://en.wikipedia.org/wiki/Weighting_factor) in information retrieval (https://en.wikipedia.org/wiki/Text_mining, and https://en.wikipedia.org/wiki/User_modeling) (Aizawa, 2003). We also used this method for feature generation to investigate their impact on offensive language detection. This method calculates the importance of a word in a document being a part of the whole corpus. In addition, it also computes the ratio of the word in the whole corpus by taking the log of total documents in the corpus divided by the number of documents in which the term appears. It is the product of term frequency and inverse document frequency. To extract TF-IDF, and n-gram features we used the scikit-learn library on a labeled corpus of 7,500 posts and comments.

#### Word embeddings

Word embedding is one of the most popular representations of text vocabulary and can capture the context of a word in a text, such as semantic and syntactic similarity and relationship with other words. Mikolov et al. (2013) developed the word2vec method to learn word embeddings. It is an unsupervised shallow two-layer neural network trained for generating high-quality, distributed, continuous dense vector representations of words. Word2vec supports two model architectures to produce a distributed representation of words, *i.e.*, continuous bag-of-words and continuous skip-gram models (https://en.wikipedia.org/wiki/Distributed_representation). In the continuous bag-of-words model, the current word is predicted from a window of surrounding context words, whereas the skip-gram algorithm predicts the surrounding window of context words using the current word. We used the skip-gram model and generated 100 dimensions.

Furthermore, to train the word2vec embedding model, we used a corpus of 196,226 Facebook posts to create embeddings for a unique vocabulary using the Genism library of Python. To the best of our knowledge, word embedding has not been used to explore and detect Urdu offensive language in the literature. We have used the word2vec feature generation method in the Urdu language and have compared its performance with word n-gram, bag-of-words, and TF-IDF methods.

### Classifiers and evaluation measures

**Figure 3:**
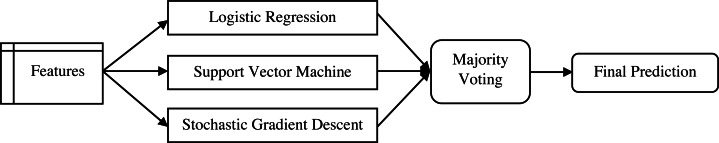
The pipeline of the ensemble model.

In this study, five ML algorithms have been selected for experimental setup, to develop a robust model for offensive language detection in Urdu. The models are logistic regression (Logistic-Reg), random forest (RF), stochastic gradient descent (SGD), support vector machine (SVM), and an ensemble model. The pipeline of the ensemble model is presented in Fig. 3. The majority voting methodology is adopted in the ensemble model and Logistic-Reg, SVM, and SGD are the three ML models being used. We compare the performance of five ML models, and the best model is defined.

 In addition, the 10-fold cross-validation method is used for model training and testing. The results have been reported using standard accuracy, precision, recall, F1-score, area under the curve (AUC), and Matthews correlation coefficient (MCC) measures.

The mathematical definitions of these metrics are described as follows:

### Accuracy

It is measured as the ratio of the number of correctly predicted instances (positive and negative) to all predictions.

(3) \begin{eqnarray*}\text{Accuracy}= \frac{\text{TruePositive}+\text{TrueNegative}}{\text{TruePositive}+\text{TrueNegative}+\text{FalsePositve}+\text{FalseNegative}} .\end{eqnarray*}

### Precision

It is the measure that summarizes the fraction of actual instances of an offensive class to the total number of instances assigned an offensive class label.

(4) \begin{eqnarray*}\text{Precision}= \frac{\text{TruePositive}}{\text{TruePositive}+\text{FalsePositive}} .\end{eqnarray*}

### Recall

It summarizes how well the offensive class is predicted and it is calculated as

(5) \begin{eqnarray*}\text{Recall}= \frac{\text{TruePositive}}{\text{TruePositive}+\text{FalseNegative}} .\end{eqnarray*}

### F1-Score

F1-score is the combination of precision and recall metrics that balances both measures. It is calculated as

(6) \begin{eqnarray*}\mathrm{F}1-\text{measure}= \frac{2\text{*Precision*Recall}}{\text{Preceision}+\text{Recall}} .\end{eqnarray*}

### The AUC

It relates the true positive rate to the false positive rate and provides an aggregate measure of performance across all possible classification thresholds.

### The MCC

It is a reliable statistical rate that produces a high score only if the prediction obtained good results in all of the four confusion matrix categories, *i.e.*, true positives, false negatives, true negatives, and false positives.

## Experimental Setup and Results Analysis

In this section, four experiments are performed. The first experiment compared the performance of five ML methods. Also, the impact of each type of feature on offensive language detection is investigated. In the second experiment, the performance of the proposed model is compared with the baseline. In the third experiment, feature selection is performed using the wrapper method and its implications are discussed. In the last experiment, the impact of several combinations of proposed features is examined and conclusions are drawn. The necessary parameters of ML models are presented in Table 3 to replicate the results.

### Experiment 1: performance comparison of Ml methods and feature models

Here experiments are conducted to meet two objectives: (1) performance comparison of five ML models using proposed features, (2) investigation of the impact of four types of features for offensive language detection in Urdu. The results are evaluated using accuracy, precision, recall, F1-measure, AUC, ROC curves, and MCC. The ML methods are trained and tested using the standard 10-fold cross-validation technique. The performance of each classifier with word unigram, TF-IDF, bag-of-word, and word2vec methods in the accuracy metric is presented in Table 4. It has been observed that the ensemble model outperforms all other classifiers. In addition, the word2vec feature model demonstrated better performance as compared to bag-of-word, TF-IDF, and word unigram. The reason behind achieving 88% accuracy with the word2vec feature is that this model employs the semantic/contextual information related to the language of posts/comments. Overall, the word unigram presented the lowest performance as compared to other feature models. On the other end, RF demonstrated the least performance as compared to the other four ML methods. In addition, it is observed that the word2vec feature model with all the classifiers demonstrated the best performance in comparison with other feature models. In contrast, we did not obtain promising results when all features are combined.

**Table 3:** Parameters of ML models.

**S #**	**Classifier**	**Parameter**	**Value**
1	Logistic Regression	Penalty	L2
2			Max-iteration	2,000
3		Random-state	42
4	Support Vector Machine	Penalty	L2
5			Random-state	42
6		Kernel function	radial
7	Stochastic Gradient Descent	Loss	Hing
8			Penalty	L2
9			Max-iteration	50
10		Random-state	42
11	Random Forest	n-estimators	10
12			Random-state	10
13		Number of trees	200

**Table 4:** Comparison of ML techniques and feature-wise performance (accuracy measure).

**Features**	**SVM**	**Logistic-Reg**	**SGD**	**RF**	**Ensemble**
Word-unigram	83.40	84.18	83.06	79.13	85.51
TF-IDF	83.64	83.63	83.44	80.17	85.95
Bag-of-word	82.89	84.36	83.17	80.29	84.94
Word2vec	87.05	86.95	84.92	81.42	88.27
All	84.24	84.78	83.64	80.25	86.16

Similarly, the performance of five ML models with four feature models using precision metric is demonstrated in Table 5. Again, the performance of word2vec is better than all other features and achieved 88.28% precision with the ensemble model. The accuracy and precision measures justify that our results are consistent along both measures, and word2vec and ensemble models are the best feature and ML models. However, predictive performance with all features is not promising using the precision measure (*i.e.*, 86.22% but 88.26% with word2vec).

**Table 5:** Comparison of ML techniques and feature-wise performance (precision measure).

**Features**	**SVM**	**Logistic-Reg**	**SGD**	**RF**	**Ensemble**
Word-unigram	83.40	84.15	83.07	79.08	85.50
TF-IDF	83.54	83.46	83.34	79.90	85.84
Bag-of-word	82.86	84.32	83.19	80.07	84.92
Word2vec	87.00	86.91	84.90	81.30	88.20
All	84.2	84.71	83.62	80.08	86.11

The performance of four feature types is also compared using the recall evaluation metric for offensive language detection. 10-fold cross-validation and five ML methods are used for experimentation. The performance of the word2vec feature model is observed to be better than word unigram, bag-of-word, and TF-IDF feature models as shown in Table 6. The best performance (88.27% recall) is achieved with word2vec and the ensemble model. On the other hand, word unigram achieved the lowest recall with the RF model and the best recall with the ensemble model. The outperformance of the word2vec feature model with the ensemble model remains consistent along with the recall measure.

**Table 6:** Comparison of ML techniques and feature-wise performance (recall measure).

**Features**	**SVM**	**Logistic-Reg**	**SGD**	**RF**	**Ensemble**
Word-unigram	83.78	84.67	83.43	79.65	85.53
TF-IDF	84.20	84.48	84.17	81.48	86.09
Bag-of-word	83.26	84.87	83.62	81.27	85.00
Word2vec	87.38	87.29	85.38	81.81	88.28
All	84.65	85.32	84.15	81.05	86.22

Tables 7 and 8 shows the performance of five ML models with four feature models, using the AUC and MCC measures. Here again, we can observe that the performance of the word2vec feature model is better than word n-gram, TF-IDF, and bag-of-word feature models. Moreover, the ensemble model presented better performance as compared to the other four ML methods as shown in Tables 7 and 8. Thus, along with six evaluation metrics, the outperformance of the word2vec feature model with the ensemble model is observed to be consistent. However, we did not obtain promising results when all features are combined.

**Table 7:** Comparison of ML techniques and feature-wise performance (AUC measure).

**Features**	**SVM**	**Logistic-Reg**	**SGD**	**RF**	**Ensemble**
Word-unigram	90.29	90.80	90.24	86.67	93.03
TF-IDF	91.01	91.02	91.03	86.28	93.33
Bag-of-word	90.04	91.03	89.91	87.04	92.84
Word2vec	91.78	91.76	90.54	87.20	95.00
All	90.78	91.15	90.42	86.79	93.55

**Table 8:** Comparison of ML techniques and feature-wise performance (MCC measure).

**Features**	**SVM**	**Logistic-Reg**	**SGD**	**RF**	**Ensemble**
Word-unigram	0.682	0.695	0.669	0.616	0.787
TF-IDF	0.704	0.683	0.691	0.619	0.790
Bag-of-word	0.660	0.704	0.654	0.605	0.783
Word2vec	0.737	0.738	0.746	0.668	0.838
All	0.695	0.705	0.69	0.627	0.795

**Figure 4:**
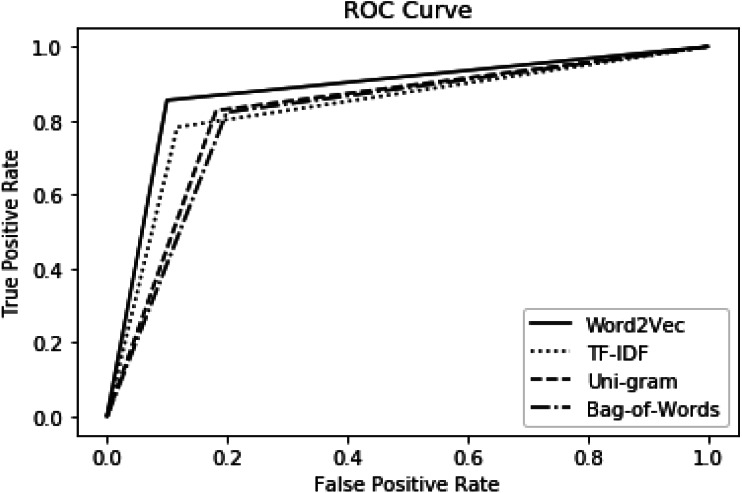
Proposed feature performance with ensemble model using ROC.

The impact of proposed features is also investigated using the ROC curve as presented in Fig. 4. For experimental setup, the ensemble model is used as a classifier and 10-fold cross-validation is used for training and testing purposes. It is depicted in Fig. 4 that word2vec demonstrates the best performance and has covered more area under the curve. The performance of TF-IDF is comparable with word2vec features but slightly lower. We find symmetry in the results of ROC and other evaluation measures. Hence, the performance of features is consistent along six metrics. Thus, this proves the significance of the word2vec feature model and ensemble model for the detection of offensive language in Urdu.

### Experiment 2: comparison with baseline

The latest work on offensive language detection in the Urdu language is presented by Akhter et al. (2020). In this section, we present a comparison of our model with Akhter et al. (2020). First, there is a difference in the nature and size of the dataset, as the baseline paper has created a dataset that was collected from comments in the Urdu language from different YouTube videos. These comments were manually collected from videos by the authors themselves. In our case, we have collected actual comments, shared by users, on 19 different Urdu websites including newspapers, political parties, *etc*. Therefore, our dataset presents originality not only in the context of comments but also in the text of the comments. Second, our dataset is much larger, starting from more than 800,000 comments, it has concluded to almost 200,000 comments after preprocessing (Table 2), whereas the baseline paper contains 2,000 comments in the Urdu language, and they have not mentioned the source of Roman Urdu. Third, Akhter et al. (2020) got the comments annotated by a panel of three persons who were all students, whereas we used the services of five language expert annotators. This has strengthened the quality of our labeling.

Regarding experiments, the baseline (Akhter et al., 2020) focused more on char and word n-gram feature extraction techniques and used several (more than 15) classifiers, whereas, in our work, we have focused on a variety of the latest feature extraction techniques and used more relevant classifiers generally used in literature for a similar task. In this context, we have performed experiments of Akhter et al. (2020) using our dataset and reported only those experiments which outperformed. Both the word n-gram-based and character n-gram-based, experiments are reported in Table 9. Our model with four feature extraction methods and ensemble as a classifier is also presented and our model has demonstrated better results as compared to the standard baseline (Akhter et al., 2020). The AUC and MCC performances are also reported. We have used six performance metrics for the comparison of results. In addition, the best score against each performance metric is also highlighted in Table 9.

**Table 9:** Performance comparison with standard baseline.

**Features**	**Accuracy**	**Recall**	**F1-measure**	**Precision**	**AUC**	**MCC**
Word Uni + bi-gram (Akhter et al., 2020)	**85.17**	**85.13**	**85.15**	**85.15**	92.49	0.778
Word Uni+bi+tri-gram (Akhter et al., 2020)	84.78	84.82	84.81	84.76	92.28	0.777
Char tri-gram (Akhter et al., 2020)	84.31	84.30	84.38	84.30	92.42	0.774
Char bi + tri-gram (Akhter et al., 2020)	83.70	83.68	83.68	83.71	92.36	0.767
Char Uni +bi +tri-gram (Akhter et al., 2020)	84.08	84.20	84.00	84.22	92.16	0.773
Bag of word	84.92	84.94	84.90	85.00	92.84	0.783
Word-unigram	85.50	85.51	85.51	85.53	93.03	0.787
TF-IDF	85.84	85.95	85.82	86.09	93.33	0.790
Word2vec	88.20	88.27	88.22	88.28	95.00	0.838

It is depicted in Table 9, that our model has outperformed the baseline along with all performance metrics. The improvement is 3.55% in accuracy, 3.68% in recall, 3.60% in f1-measure, 3.67% in precision, and 2.71% in AUC. One important point in Table 9 is that the performance of the baseline technique on our dataset is less than those reported in their paper (Akhter et al., 2020). The main reason for this is the enormity of our dataset. As mentioned above, their dataset does not contain sufficient variation or originality due to how it has been generated by the authors themselves, by watching different YouTube videos. On the other hand, our dataset is made by collecting genuine texts shared by thousands of different people. This brings much more originality and variation to our dataset.

**Figure 5:**
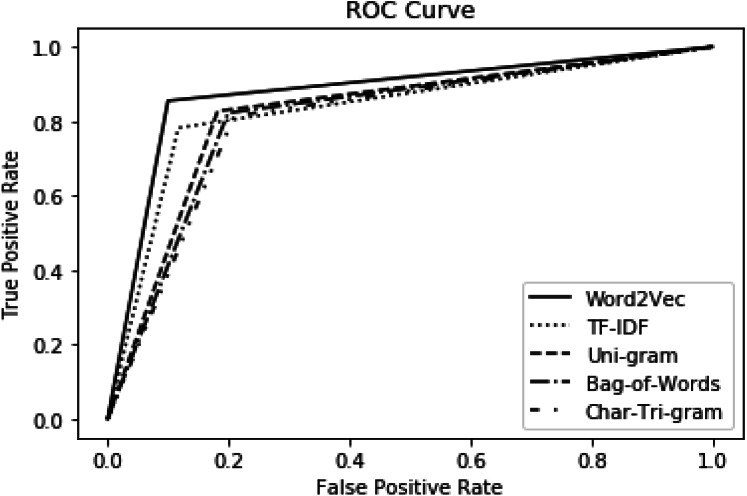
Performance comparison with standard baseline using ROC.

The comparison of our model with the baseline is also evaluated using the ROC curve as presented in Fig. 5. The performance of the baseline is represented by the char tri-gram in Fig. 5 because, in the baseline approach, the char tri-gram presented the best performance, as shown in Table 8 upper part. This is exactly the reason for its being selected as the baseline method. It has been shown clearly that word2vec presented better performance as compared to baseline and the other three types of features. Regarding our method, the worst performance is noted by bag-of-words, but it is still better than the baseline method. This proves the significance and effectiveness of our approach as compared to the baseline.

### Experiment 3: impact of feature selection on classification performance

To reduce the complexity of feature models and to enhance the performance of the proposed detection model for offensive language, we employed the technique of feature selection (Atlam et al., 2020). A well-known feature selection method is selected to find the best subset of features. *i.e.*, wrapper method. This method employs a search strategy (forward selection) by evaluating the possible subsets of features, using a machine learning algorithm. The evaluation of each subset is based on the quality of performance produced by the selected machine learning algorithm. The evaluation criteria may be any performance metric depending upon the nature of the problem. In our case, we have selected the SVM algorithm with an accuracy metric for the best subset selection. The reason behind choosing SVM is that it presented a significant feature subset while testing each ML model in the wrapper selection method. In the word unigram feature model, we have compared the performance of the selected subset with all Uni-gram features and did not find any improvement in the accuracy. Therefore, we did not consider the results of the word unigram.

After employing the wrapper method, we found the best subset of 67 features for TF-IDF, the subset of 72 features for bag-of-word, and the subset of 72 features for the word2vec model. Regarding performance using the accuracy metric, it is clearly shown in Table 10 that we found significant improvement in performance with the selected features for each type of feature. For example, the largest improvement is observed for the bag-of-word feature model whereas word2vec demonstrated a small improvement. An ensemble method with 10-fold cross-validation is used to generate the results.

**Table 10:** Impact of feature selection on offensive language detection using accuracy, precision and AUC metrics.

Features	Accuracy measure		Precision measure		AUC measure	
	Without	With Selection	Without	With Selection	Without	With Selection
TF-IDF	85.84	86.82	86.09	87.65	93.33	94.82
Bag-of-word	84.92	87.410413	85	88.185	92.84	96.04
Word2vec	88.2	88.47	88.28	88.52	95	95.95

It is clear from Table 10 that feature selection impacts all performance metrics. If we compare the result precision-wise; the bag-of-words achieved the best improvement of 3.74%, as compared to TF-IDF and word2vec with improvements of 1.81%, and .27% respectively. Similarly, the accuracy and AUC measures also improved with feature selection. Furthermore, like precision, the maximum improvement is observed with bag-of-words using accuracy and AUC metrics. These provide evidence of the usefulness of feature selection in the proposed methodology.

### Experiment 4: impact of hybrid combination on classification performance

In the previous experiments, we presented the performance of the proposed features as a standalone model. In this section, we have conducted experiments to investigate the impact of various combinations of the proposed features for offensive language detection. Among four feature sets in the prior experiments, we found that word2vec has consistently outperformed the rest (with and without feature selection), whereas the performance of word unigram is the lowest among all. Ignoring the lowest one, we have made several combinations of the rest of the three feature sets. An ensemble model with 10-fold cross-validation and six evaluation metrics is used for the experimental setup.

The best performance in accuracy measure (89.23%) is achieved when we combine all three features as shown in Table 11. Similarly, this combination demonstrates the best performance on precision, recall, f1-measure, AUC, and MCC measures as well. Hence, it is the best performance obtained by our proposed model. In addition, we have observed that the second-best performance is achieved by combining bag-of-word with word2vec. The third-best performance is observed by using the combination of TF-IDF with word2vec as presented in Table 11. Thus, we can conclude that word2vec is the best feature method not only as a standalone model but also achieved the best performance when used with other features in combination. Although all evaluation metrics presented very promising values, AUC (96.78) value is very significant. Thus, the combination of the features proved its significance for offensive language detection in Urdu.

#### Examples of offensive and not-offensive posts

After an exhaustive evaluation of the proposed model, we have added here the predicted class labels of six randomly selected posts/comments from the test set as shown in Table 12. If we analyze the language of comments 1 and 2, we can conclude that the presence of offensive words might have made the proposed model label these posts as offensive. However, comment 3 does not contain any offensive words but it is the context of the comment that has guided the proposed model to declare it as offensive. In addition, a similar trend is also observed for not-offensive comments/posts labeled by the proposed model. The system labeled comments 4 and 6 as not offensive because there is no offensive word in both comment. However, the class label of comment 5 is decided as not-offensive using the context of the text available.

**Table 11:** Performance of hybrid combinations using five evaluation measures.

**Feature Combinations**	**Accuracy**	**Precision**	**Recall**	**F1-score**	**AUC**	**MCC**
Bag-of-word +TF-IDF	87.82	88.42	87.9	87.67	96.05	0.856
TF-IDF +Word2vec	88.60	88.79	88.71	88.41	95.00	0.838
Bag-of-word +Word2vec	89.00	89.50	89.20	88.93	96.01	0.849
Bag-of-word +TF-IDF +Word2vec	89.23	89.94	89.49	89.04	96.78	0.895

**Table 12:** Examples of predicted labels.

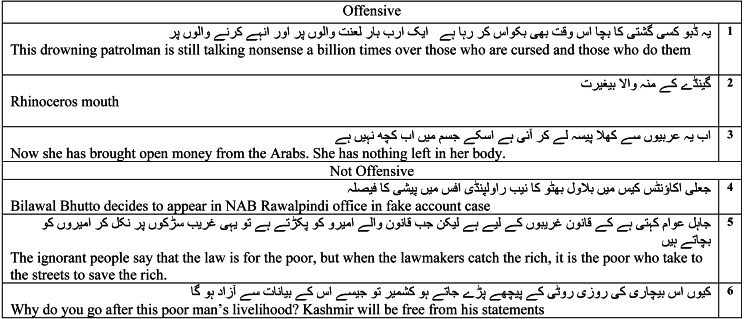

## Discussions and Implications

In recent years, social media is getting popular in every aspect of life and offensive language has become the dark side of this technology. The offensive language in social media causes extremism and intolerance, that pressurizes vulnerable groups, such as religious minorities, social activists, religious scholars, political leaders, *etc*. The findings of this research aid to uncover a more influential set of features that are supportive of offensive language detection in Urdu on the Facebook platform. This research has valuable insights for online users, website owners, and law and enforcement agencies to identify offensive language on websites. It is highly desirable to detect this type of material from social media to reduce various crimes in society. In this perspective, we have developed a detection model for offensive language in Urdu by utilizing more effective semantic and word embedding features with the ensemble method. This proposed model is tested on a real-life annotated Facebook posts dataset to find the real insights for this research to make it more practical. The results provide evidence of our detection model is effective in detecting offensive language in Urdu. We have evaluated the impact of robust features as a standalone model and as a hybrid combination with an ensemble model. The most significant contribution is the embodiment of the detection model that achieves 90% accuracy and improves 5% accuracy as compared to the baseline. Based on these findings, our proposed model can be utilized by any organization to identify offensive content in the Urdu language.

In addition, our corpus has various unique aspects. First, it covers many categories, *e.g.*, religious, political, news, regional, ethnic, vulgarity, *etc*. To the best of our knowledge, it is the first Urdu offensive language corpus that covers so many categories of offensive language using Pakistani social media platforms. Second, our annotated dataset has a higher number of offensive posts/instances as compared to not-offensive posts *i.e.*, 51% offensive instances and 49% not-offensive. The inter-annotator agreement measure is 67% which is comparable with other studies. A related survey (Fortuna & Nunes, 2018) shows that the existing datasets consist of a very low number of offensive/hate instances as compared to not hate/offensive instances. Third, it is observed that in our dataset, 27% of offensive posts consist of vulgar words, 22% of offensive posts contain sectarian words, 5% of posts have regional offensive words, and 30% of posts consist of ethnic words.

In addition, our research draws various practical implications. The outcome of this research can be used to develop a filter for online platforms of social media to early identify and discard offensive/unwanted material. It is also observed that offensive language has a strong relation with events occurring throughout the year. We found a lot of religious offensive words in comments during religious events such as Moharram and Eid Milat-n-Nabi, *etc*. Similarly, the political parties’ public pages have many comments which incite offensive language toward their opposite political leaders. Although we did not annotate the type of target, however, we found that there are many comments and posts which incite offensive words against popular political leaders, religious scholars, and human rights personnel. Thus, a fine-grained annotation of the targets of the offensive posts can be done. This enrichment may facilitate government organizations and social media platforms to identify and remove various types of offensive language from social media.

## Conclusion and Future Work

In this case study, the objective was to design a binary classification model to identify offensive content in the Urdu language. To meet this challenge, a new corpus was constructed containing Urdu posts and comments from various popular Pakistani Facebook pages. The corpus was annotated by five domain experts and the final dataset is about 7,500 instances. In contrast, the dataset used by the baseline was comparatively small. In addition, four types of feature extraction methods are utilized to generate semantic and word embedding features. The methods are word n-gram, bag-of-words, TF-IDF, and word2vec-based word embeddings. Five popular ML methods with 10-fold cross-validation and six state-of-the-art evaluation metrics are used for the experimental setup. The baseline study used only the word n-gram and char n-gram features. The findings of this study reveal that word2vec outperformed the other three types of features and standard baseline as a standalone model and achieved 88.20% accuracy. In addition, to improve the proposed framework accuracy, feature selection is incorporated using the wrapper method. We observed improvement in all evaluation metrics and classification accuracy improved significantly. The ensemble model demonstrated the best performance as compared to other ML methods. In addition, we compared the performance of different combinations of features and concluded that any combination of features with the word2vec method shows optimal performance.

There are a few avenues for future work. The latest contextual feature methods and NLP techniques may be used to improve the accuracy of the proposed model. Another direction is to utilize a Rule-based approach to handle the problem of offensive language identification. Regarding ML models, deep neural networks and evolutionary algorithms can be applied to develop more robust offensive language detection models. Similarly, the proposed methodology can be employed for other related problems in similar domains.

## APPENDIX A: Annotation Guidelines to annotate an Urdu post

This document contains all the necessary information, including offensive language definition, its types, as well as examples of how to annotate a post/comment. It was provided to the team of five human annotators.

**Offensive language types**

**1. Offending/Insulting**

**Offensive** –If a post/comment contains offending/insulting words in the context to humiliate/hurt any human being then it is labeled as Offensive. Sometimes, the offending/insulting words are being used but they are not in the context to hurt anyone.

**Not offensive** –Otherwise

The examples of offensive and not offensive posts containing insulting/offending words are provided below:

**2. Offensive reference to sex or bodily functions**

When a post/comment consists of explicit references to sex or body functions to humiliate someone then it is labeled as offensive. The examples are provided next:

**3. Sexual orientation, gender**

We label a post/comment as offensive when it contains insulting words regarding sexual orientation and gender to hurt/humiliate someone. Examples are given below:

**4. Animal names as offensive words**

In Pakistani culture, whenever a person uses animal names to address a person it is considered as offensive therefore annotate all the comments/posts as offensive where animal names are used to address humans, or groups. For your guidance consider the following examples.

## Additional Information and Declarations

### Data Availability

The following information was supplied regarding data availability: The Dataset-TF-IDF is available at figshare: Iqbal, Shahid (2022): dataset-tfidf20.xlsx. figshare. Dataset. https://doi.org/10.6084/m9.figshare.20347197.v1
The Dataset-word2vec is available at figshare: Iqbal, Shahid (2022): dataset-word2vec features.xlsx. figshare. Dataset. https://doi.org/10.6084/m9.figshare.20347221.v1
The Python final data extraction is available at figshare: Iqbal, Shahid (2022): finaldataextraction.py. figshare. Online resource. https://doi.org/10.6084/m9.figshare.20347227.v1
The Python final implementation is available at figshare: Iqbal, Shahid (2022): finalimplementation.py. figshare. Online resource. https://doi.org/10.6084/m9.figshare.20347233.v1. The data and code are available at Zenodo: Sajid Hussain, Muhammad Shahid Iqbal Malik, & Nayyer Masood. (2022). Offensive content dataset in Urdu language (Version V1) [Data set]. Zenodo. https://doi.org/10.5281/zenodo.7207438.
